# Non-Invasive UWB Sensing of Astronauts' Breathing Activity

**DOI:** 10.3390/s150100565

**Published:** 2014-12-30

**Authors:** Marco Baldi, Graziano Cerri, Franco Chiaraluce, Lorenzo Eusebi, Paola Russo

**Affiliations:** Università Politecnica delle Marche, Dipartimento di Ingegneria dell'Informazione, Via Brecce Bianche 12, Ancona 60131, Italy; E-Mails: m.baldi@univpm.it (M.B.); g.cerri@univpm.it (G.C.); lorenzo.eusebi.pro@gmail.com (L.E.); paola.russo@univpm.it (P.R.)

**Keywords:** breath detection, electromagnetic compatibility, sensors, radiated emission, radiated susceptibility, signal processing, space missions, spectral analysis, ultra wide bandwidth

## Abstract

The use of a UWB system for sensing breathing activity of astronauts must account for many critical issues specific to the space environment. The aim of this paper is twofold. The first concerns the definition of design constraints about the pulse amplitude and waveform to transmit, as well as the immunity requirements of the receiver. The second issue concerns the assessment of the procedures and the characteristics of the algorithms to use for signal processing to retrieve the breathing frequency and respiration waveform. The algorithm has to work correctly in the presence of surrounding electromagnetic noise due to other sources in the environment. The highly reflecting walls increase the difficulty of the problem and the hostile scenario has to be accurately characterized. Examples of signal processing techniques able to recover breathing frequency in significant and realistic situations are shown and discussed.

## 1. Introduction

The possibility of using ultra wide bandwidth (UWB) technology for monitoring some physiological parameters, for example, concerning the respiration activity of astronauts, has been the objective of the project entitled “Non Invasive Monitoring by Ultra wide band Radar of Respiratory Activity of people inside a spatial environment” (NIMURRA), funded by the Italian Space Agency (ASI). The project has involved four Universities, namely: “La Sapienza” University in Rome, University of L'Aquila, University of Bologna and Polytechnical University of Marche, and two Italian small/medium enterprises, Advanced Computer Systems (ACS) and Kayser Italia, whose main core business is the development of systems in support of space missions.

The chance to estimate in real time the frequency and depth of respiratory acts is of particular importance [1], because, as the environment pressure varies, compensation mechanisms can be triggered, which indirectly highlight a possible suffering state of the organism because of a lack of oxygen. Despite subjective symptoms are extremely variegated, a modification of both frequency and depth of respiratory acts is always present, and can be considered as a marker in case of hypoxic suffering (lack of oxygen). It is evident that in such conditions it would be extremely useful to be able to monitor the respiratory activity of the astronauts, an investigation which cannot be carried out by means of the classical optoelectronic plethysmography technique [2] both for the limitations that the needed petticoat would impose and because this technique requires immobility from the patient. Starting from the previous considerations, the rationale of the project for sensing the respiratory activity concerned the design of a completely non-invasive technique, which also allows the subject under examination to freely move.

The monitoring of the astronauts can be performed in different environments and situations, for example when they are working inside the space station modules, during the extra vehicular activity (EVA), or when they are resting. In particular, the project focused on the monitoring of the astronauts when they are inside the crew quarter devoted to rest. In fact, rest and sleep have been consistently reported as being of poor quality in microgravity, both by American astronauts and Russian cosmonauts [3,4], but up to now only few studies have addressed the combined action of microgravity and rest on respiration and chest wall mechanics.

Radio frequency (RF) systems for the detection of the physiological activities have been proposed in the literature, but, to the authors' knowledge, they were never specifically addressed for aerospace applications; such equipment can be grouped into two main families: Doppler and UWB systems. Historically, Doppler systems were considered first for the detection of vital signs. The idea consists of revealing thorax movements to obtain the respiratory frequency using radar techniques in the microwave region [5–10]. Most of the research effort is devoted to the processing of the received signal, and several techniques which make the processing more robust were described and implemented [11,12] to investigate also the capability to detect heartbeat. More recent papers are essentially addressed to overcome existing difficulties for applications to realistic environments, introducing more effective processing strategies [13,14], whereas other works deal with practical aspects related to the electronic device fabrication [15–17].

A different choice is offered by the capability of UWB radars to pass through obstacles, by their high resolution, low costs, low energy consumption, low power spectral density levels, associated with ultra-short pulse transmission and immunity against multipath interference [18]. The first applications to cardio-respiratory activity monitoring of UWB radar have been proposed by McEwan in [19], where a baseband radar with “range gating” receiver is proposed. The features of the UWB radar were also exploited to extract human vital signs in complex environments [20–23], whereas specific UWB radar systems [24–27] were designed for the rescue of victims trapped in rubble.

The novelty of the NIMURRA project is represented by the application of the UWB technique to monitor astronauts' respiration in a space environment. Despite the premises above, in fact, the use of a biomedical radar in this involved scenario has not been extensively studied in previous literature. This very specific contest requires an accurate electro-magnetic (EM) characterization of the module. More precisely, two aspects of paramount importance for the system design are analyzed in this paper. The first aspect regards the compatibility of the system with the surrounding environment in terms of emitted electromagnetic noise, because both the UWB system and external equipment can be considered at the same time culprit and victim of malfunctioning. As a consequence the analysis of robust algorithms to cancel out interfering signals for a correct detection of the breathing frequency is the second mandatory requirement.

The installation of equipment on board the International Space Station (ISS), that could be assumed as a valuable testbed for the application of the proposed system, involves a detailed analysis of the electromagnetic compatibility (EMC) aspects. The importance of this issue is proved by previous experimental analysis on airplanes [28–30] where emission of UWB signals have been evaluated within the bandwidth of some aerospace electronics. Therefore, at first, we show how some design constraints have been assessed in order to take into account the presence of other electronic equipment already on board of the station, to avoid any undesired electromagnetic emissions which may cause malfunctioning of other devices (emission issues) and to withstand different radiated and conducted emissions from other sources (immunity issues).

However, compliance with electromagnetic interference (EMI) international standards is not the only requirement, because the capability of the system to recover the correct breathing frequency has to be assured also in the presence of low level disturbing signals, both narrow band and broad band, superimposed on the received pulses. Suitable signal processing algorithms must be developed for such a purpose. Conceptually, the breathing rate can be obtained by means of a Fourier analysis, that is, a fast Fourier transform (FFT), if necessary through effective implementations like the chirp-Z transform (CZT), which allows to isolate the fundamental frequency of an “essentially periodic” signal as the breath is (at least in normal conditions). The fundamental frequency corresponds to identifying the position of the major peak in the spectrum. However, the correct identification of the breathing rate should occur, as mentioned, in a noisy environment and in the presence of reflected signals due to obstacles and objects around the monitored astronaut. This may require the adoption of smart numerical tools and procedures, for example consisting of the adoption of multi-peak detection [31] or multicolumn FFT [32]. Although we have also taken into account these variants, the results we will show throughout the paper will be mostly referred to the classical approach, which consists of searching for the major peak only. In fact, such a method is often sufficient for estimating the breathing rate, even in the complex space environment considered. Moreover, in this paper, besides the evaluation of the breathing rate, another target is the reconstruction of the entire breathing signal. This goal has been pursued as well, through the employment of a suitable reference signal. We have also faced the problem of compensating possible random movements during rest, by introducing suitable tools in the signal processor. Both the EM and signal processing approaches have been validated through laboratory experiments in order to assess the reliability of the simulation tools that have been used to model the complex and critical space applications.

The paper is organized as follows: in Section 2 a brief description of the whole system is reported; in Section 3 the system compliance to international EMI standards is addressed; in Section 4 the algorithms for the signal processing are presented; in Section 5 significant results concerning the application of the algorithms to a signal degraded by environmental electromagnetic interference are reported; finally some conclusions are summarized in Section 6.

## 2. System Description

**Figure 1.**
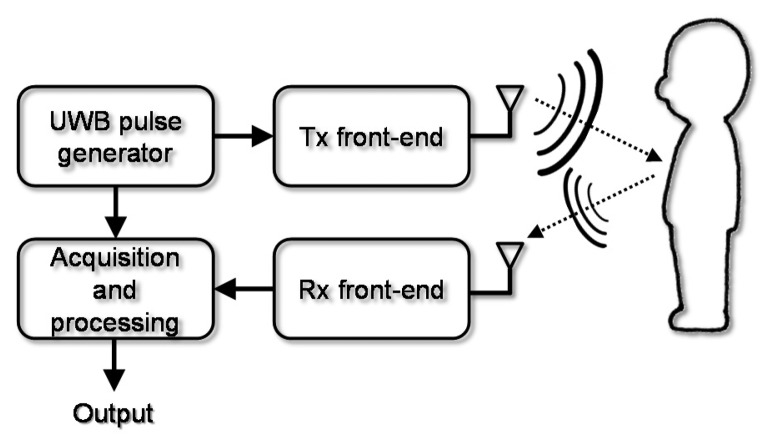
Block diagram of the considered sensing system.

The proposed monitoring system is based on the measurement of the signal reflected back by a subject when radiated with a UWB antenna system. The basic elements of the proposed architecture are shown in Figure 1. The UWB pulse generator sends a train of ultra-short pulses to the transmitter front-end, which is terminated with the transmit antenna. The antenna modifies the shape of the pulses (this must be taken into account in the design) according to its transfer function characteristics. The pulses are reflected by the moving thorax of the subject and come back to the receive antenna where they are acquired and sent to the acquisition and processing system, which gives in output the desired unknown quantities, e.g., the breath frequency.

More precisely, the system radiates different pulses with a pulse repetition frequency (PRF) high enough to sample accurately the thorax displacements. During the respiration activity the thorax of the subject changes its position relative to the antenna and so the reflected pulses arrive at the antenna with different delay times. Based on the measurement of such delays, the breathing frequency and the time evolution of the respiration can be recovered with good accuracy through a suitable processing of the received waveforms. Such a procedure, requires the individuation and definition of the following parameters [33]: (1) Level, profile, and temporal duration of transmit-receive pulse; (2) Position and width of the transmit-receive antenna beam; (3) Re-irradiation levels and patterns caused by the subject experiencing the EM field action; (4) Levels of power absorbed in the different organs of the exposed subject; (5) Algorithms to extract data of interest from the received signal and to elaborate them.

The adoption of a UWB sensing system in the considered context is justified by the several advantages it offers with respect to more conventional solutions. Besides the fundamental feature to be completely non-invasive, that was a pre-requisite of the NIMURRA project, further advantages of the UWB technique related to the very short pulses (and, consequently, very large bandwidths) involved are: high spatial resolution, high sensitivity (by using the so-called range gating technique), low values of power spectral density, almost negligible risk of undesired biological effects on the monitored subject, and low cost. Moreover, the UWB pulses are not influenced by blankets or clothes.

A common choice for the pulse shape is to use the *n*-th derivative, *n* ≥ 1, of the Gaussian function. By centering the pulse at the time instant τ = 0, we have:

(1) $$p_{n}(\tau )=C_{n}{\left(-1\right)}^{n}\frac{1}{{\left(\sigma_{n}\sqrt{2}\right)}^{n}}H_{n}\left(\frac{\tau }{\sigma_{n}\sqrt{2}}\right)G\left(\tau ,\sigma_{n}\right)$$

where *C_n_* is a constant determining the pulse amplitude, *p_n_*(τ) is the pulse waveform as a function of the time τ, *H_n_* is the Hermite polynomial of order *n* and *G* is the Gaussian function with zero mean and standard deviation σ*_n_*. The latter determines the effective pulse duration. For example, in the case of *n* = 1 (Gaussian monocycle), σ*_n_* = τ*_p_*/2, where τ*_p_* is the time gap between the maximum and the minimum of the monocycle. As the pulses so defined have nominally infinite extension in time, their effective duration τ*_d_* must be properly defined. For example, we can consider the interval outside which the pulse assumes, in modulus, values not greater than 1‰ of the maximum amplitude. In the case of *n* = 1, this corresponds to have τ*_d_* ≈ 3.75τ*_p_*. Finally, for the same case of *n* = 1, we have τ*_p_* = 1/(π*f_c_*), where *f*_c_ is the frequency at which the spectrum of the pulse is maximum (central frequency). The UWB signal spectrum has a central role in the analysis and will be discussed in Section 3.

The antenna has to work in the 3.1–10.6 GHz band according to the US Federal Communications Commission (FCC) definition [34]. During the project, different types of antenna have been analyzed mostly based on the Vivaldi antenna structure, because it provides the required large bandwidth and a high fidelity factor [35]. Results, not reported here for the sake of brevity, show that the system performance is not significantly affected by the antenna type. Therefore, the only antenna we have considered for the following analysis and simulation is an antipodal Vivaldi antenna.

**Figure 2.**
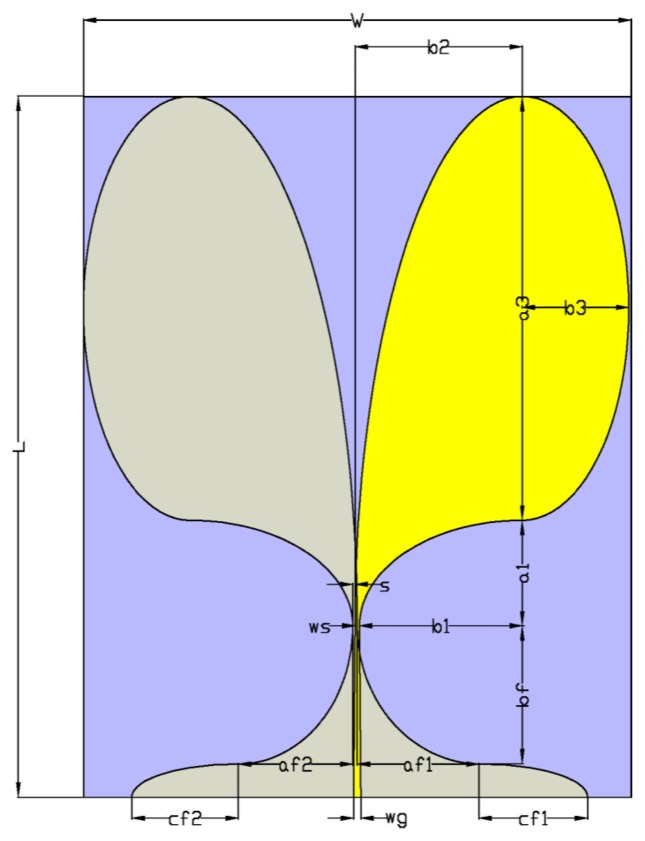
Antipodal Vivaldi antenna.

The antenna (Figure 2) has been designed and realized in our laboratory, following a proper optimization by a commercial software [36]. The required design specifications consist of a half power beamwidth (HPBW) in the order of 55° to illuminate uniformly the thorax at a distance of 50 cm, a fidelity factor > 0.9, and an S_11_ < 10 dB in the UWB band. The geometry of the realized antennas is shown in Figure 2. The final dimensions of the antenna are *L* = 171 mm, *w* = 114 mm, and the fidelity factor is equal to 0.978 in the main lobe direction.

**Figure 3.**
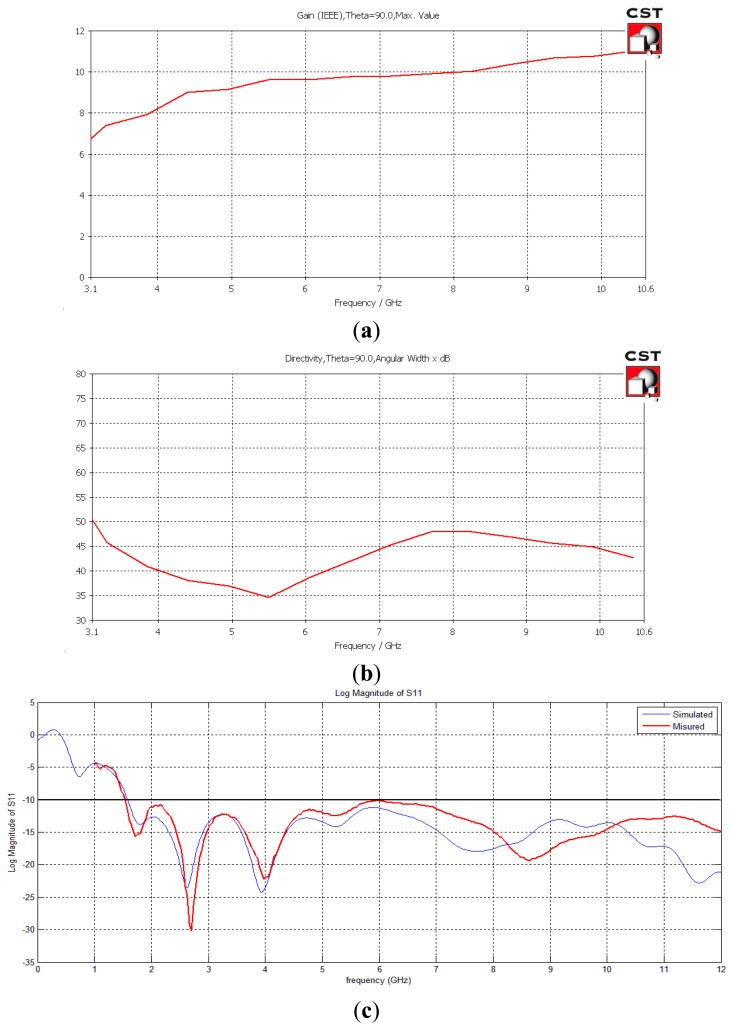
(**a**) Gain; (**b**) HPBW; (**c**) Comparison between the measured and the simulated S_11_ of the designed Vivaldi antennas.

The fundamental features of the antennas, that is, gain, HPBW and S_11_, respectively, are reported in Figure 3. The electromagnetic environment analyzed in the paper can be considered stressing for the EM simulator and therefore some measurements were carried out to check the proper setting of the parameters and the corresponding accuracy of the numerical results. The first of such tests concerns the measurement of the characteristics of the designed antennas that was performed with a vector network analyzer (VNA). In particular the S_11_ was measured, because it is one of the most critical parameters of an antenna. The results of the S_11_ measurements are reported in Figure 3c, and there compared with the simulations. As we can see, the agreement between measurements and simulations results is very good.

**Figure 4.**
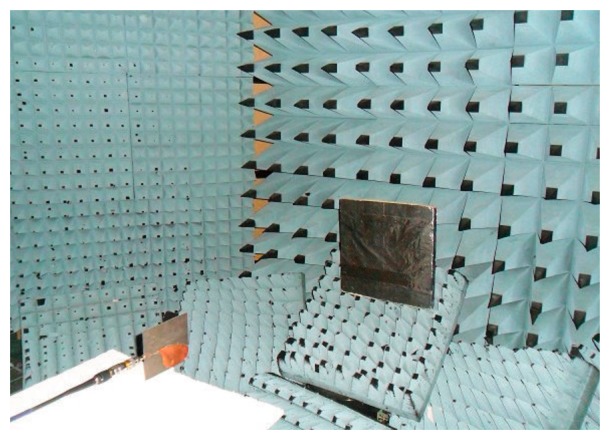
Photo of the setup considered for the software stress test.

**Figure 5.**
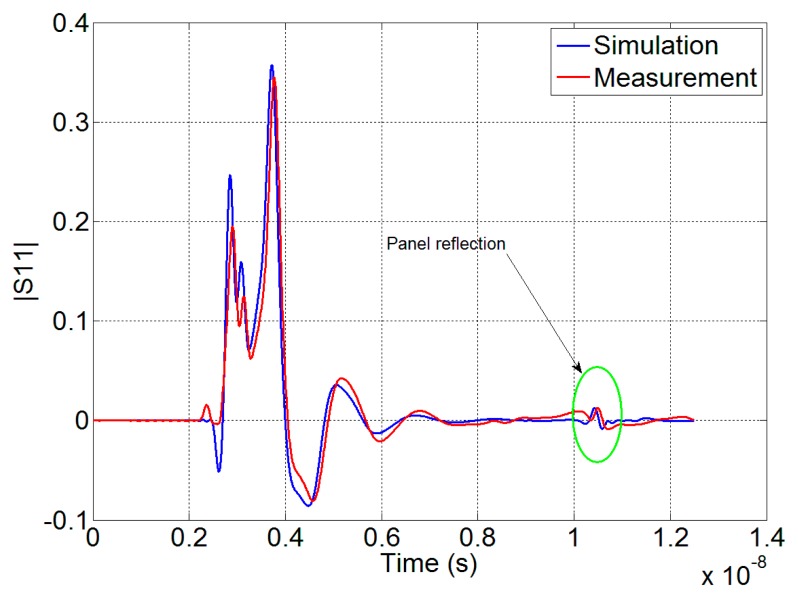
Reflection of the metallic panel: comparison between the simulation and the measurement.

Another configuration taken into account for the software stress test is shown in Figure 4, where the antenna is placed in front of a metallic panel (40 cm × 40 cm) and the S_11_ of the antenna was measured and simulated. The panel is placed in the near field region of the antenna at a distance of 100 cm. The measurements were carried out inside an anechoic environment. In Figure 5 the signal received by the antenna is reported for both the simulation and the measurements, showing a good agreement. The time domain response of the system to a pulse generated by the VNA is reported: the highest peaks in the range [2–7] ns represent reflections due to the antenna and connectors mismatching, even if we have measured |S_11_| < −10 dB in the whole UWB range. Also in this case the comparison is very good. This means that even if the antenna mismatch is not high in absolute value, it has to be compared with the useful signal that can be even lower. It is worth noticing that the reflection of the metallic panel is a very low signal that may be masked by environmental disturbances.

## 3. EMC Issues

The characterization of the environment from an electromagnetic point of view plays an important role for the definition of the UWB radar emission and susceptibility specifications. In particular we have decided to refer to the worst combination between the EMC requirements fixed for two important space module laboratories: Columbus [37] and Harmony (also known as Node 2) [38], both present in the ISS.

### 3.1. Compliance with Standard for Emissions

**Figure 6.**
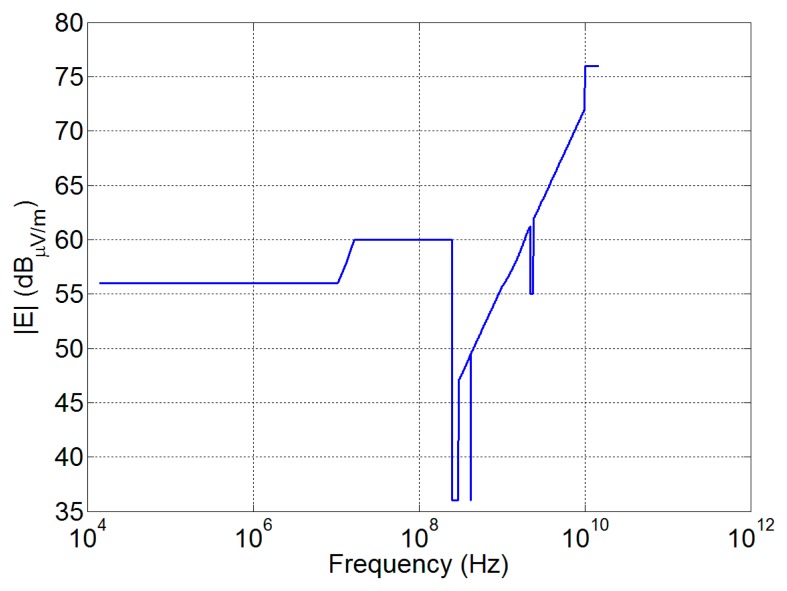
Electric field limits as a worst-case combination of Columbus and Harmony EMC requirements.

Radiated emission (RE) measurement procedures are defined by the MIL-STD 461 [39] and 462 [40], and the maximum allowed radiated emission levels are reported in Figure 6. As the measurement distance from the antenna is fixed at 1 m, the far field antenna behavior cannot be assumed for the RE prediction and a field computation in near field is required.

The aim of the analysis is the assessment of the design specification concerning the maximum input voltage feeding the antenna that allows system compliance with the emission standards. For such purpose, the field radiated by different kinds of antenna (a double ridge antenna, a printed Vivaldi antenna, and a solid Vivaldi antenna) has been analyzed, using the CST software [36] to test the robustness of the approach with respect to the radiating systems. In particular, the relationship between the allowed electric field limits, the pulse spectral density and the pulse waveform has been determined.

As the EMC standards are given in the frequency domain, the transfer function *H*(*f*) between the Fourier transform of the 1 m electric field, *E*(*f*), and the Fourier transform of the antenna input voltage, *V*(*f*), is computed:

(2) $$\begin{array}{cc} H(f)=\frac{E(f)}{V(f)} & \left[{\text{m}}^{-1}\right] \end{array}$$

This quantity is used to recover the voltage spectral density limit *S*(*f*) for the UWB pulse applicable to the antenna, in order to fulfill the electric field limits *E*_limit_ previously shown in Figure 6:

(3) $$\begin{array}{cc} \left|S(f)\right|=\frac{\left|E_{\text{limit}}\right|}{\left|H(f)\right|\cdot \text{RBW}} & \left[\text{V}/\text{Hz}\right] \end{array}$$

where RBW is the resolution bandwidth of the measurement instruments.

The emission level measured with the standard procedure depends on the PRF of the UWB signal compared to the RBW of the instrument. In Equation (3) we assume that PRF ≪ RBW, so that the emission level is simply the inverse of the RBW of the instrument.

**Figure 7.**
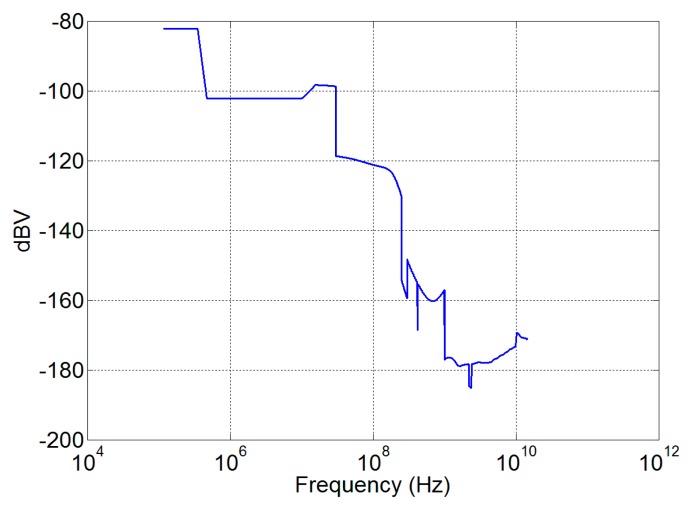
Spectral density limits for the analyzed antenna input voltage. RBW = 1 MHz ≫ PRF.

Figure 7 reports |*S*(*f*)| from Equation (3) when RBW = 1 MHz (as prescribed by standard) for the considered Vivaldi antenna. This figure provides the root mean square (RMS) value of an upper limit to the spectral density of the admissible pulse in its single side representation. In this sense, it allows to check the applicability of a UWB pulse.

Considering the pulse described in Equation (1), the corresponding spectral density is:

(4) $$P_{n}(f)=C_{n}\cdot \sqrt{2}\cdot {\left(-j2\pi f\right)}^{n}\cdot G^{\prime }(f,\sigma_{n})$$

where *C_n_* is the unknown amplitude, *G*′(*f*, σ*_n_*) is the Fourier transform of the Gaussian function and the factor $\sqrt{2}$ accounts for the single side representation and for the RMS calibration of the peak detector. For example, considering the first five time derivatives of the Gaussian pulse with σ_1_ = σ_2_ = 75 ps, σ_3_ = 60 ps, σ_4_ = 55 ps, σ_5_ = 50 ps, that ensure an almost equal pulse duration, it is possible to recover the greatest value of the amplitude *C_n_* in Equation (4) that fulfills the emission limits.

**Figure 8.**
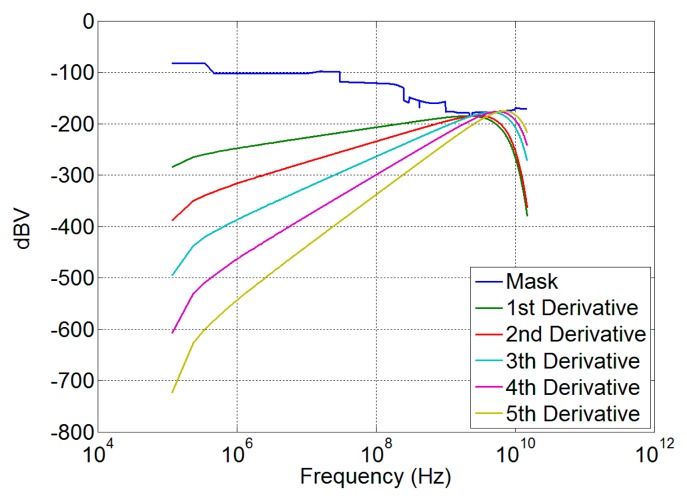
Admissible spectral density for the exciting pulses.

**Figure 9.**
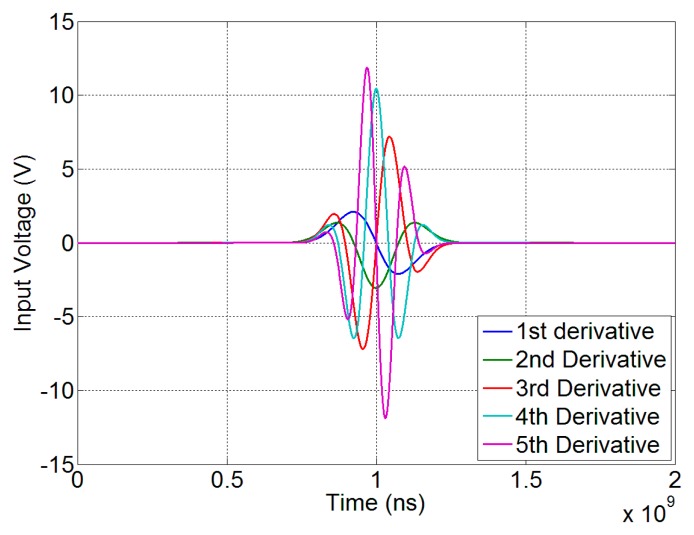
Pulse time evolution for the first five derivatives of the Gaussian pulse.

Figure 8 shows the spectral densities of the UWB input signals corresponding to the time impulse reported in Figure 9. It is noticeable that the deep notch at 2.45 GHz produces the highest limitation in the pulse amplitude up to the third derivative, whereas for higher order derivatives its effect is less evident.

### 3.2. Compliance with the Standard for Susceptibility

**Figure 10.**
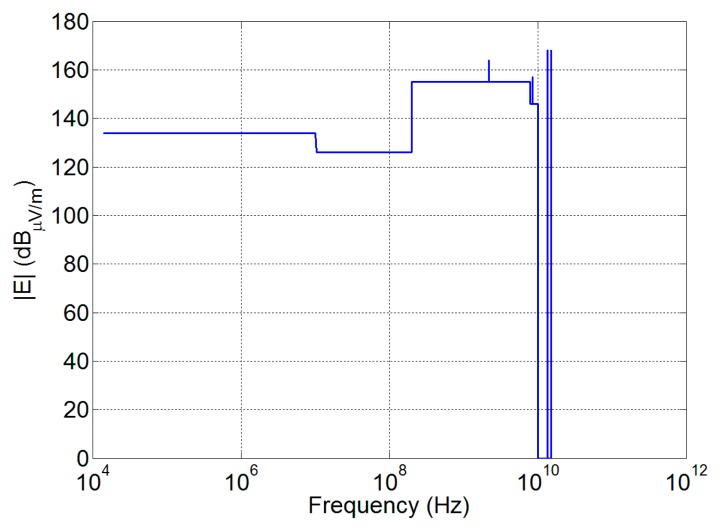
Susceptibility levels considered during the simulation, obtained as a worst-case combination of Columbus and Harmony EMC requirements.

Susceptibility assessment is considered according to the procedures standardized by MIL-STD 461 [39] and 462 [40], and the mask shown in Figure 10 that represents the worst combination between the Columbus and the Harmony EMC requirements.

In order to design an immune device, the voltage received by the antenna during a susceptibility test has to be calculated. Considering that the device has to be uniformly radiated, an incident plane wave is used. The electromagnetic configuration of the susceptibility test is different with respect to the emission test: in particular the former is defined for far field conditions, whereas the latter has to be obtained in a near field situation. As a consequence, Equation (2) cannot be used, and a new transfer function *R*(*f*) for the antenna in receiving mode has to be evaluated. Moreover, the modulus only is taken into account because the standards only specify the field intensity; then, we have:

(5) $$\begin{array}{cc} \left|R\left(f\right)\right|=\frac{\left|V\left(f\right)\right|}{\left|E\left(f\right)\right|} & \left[\text{m}\right] \end{array}$$

where |*E* (*f*)| is the amplitude of the plane wave impinging on the antenna, and |*V* (*f*)| is the corresponding maximum amplitude of the received voltage. From Equation (5), the susceptibility voltage level for each frequency is:

(6) $${O\left(f\right)|}_{{\text{dB}}_{\text{V}}}={M\left(f\right)|}_{{\text{dB}}_{\text{V}/\text{m}}}+{R\left(f\right)|}_{{\text{dB}}_{\text{m}}}$$

where *M*(*f*) is the standard susceptibility field level, and *O*(*f*) its corresponding received voltage.

**Figure 11.**
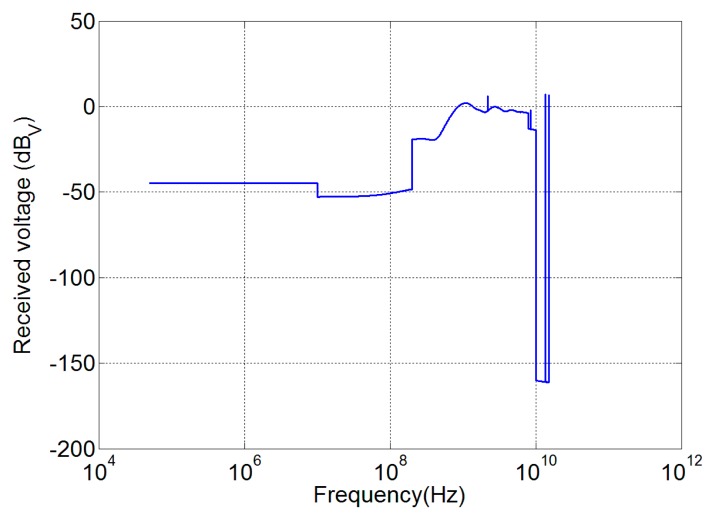
Susceptibility voltage level for the Vivaldi antenna.

As an example of the procedure, the transfer function of the Vivaldi antenna has been calculated and the received voltage, corresponding to the electric field of Figure 10, has been recovered. The result obtained is shown in Figure 11. This voltage can be used by the designer of the sensing system receiver to verify the front end components hazard and to establish proper countermeasures (limiters or suppressor devices for example).

## 4. Signal Processing Solutions

In this section, we describe the theoretical and software tools we have adopted for extracting the respiration features and, in particular, the breath frequency from the received signal. Some preliminary numerical examples will be also provided, that however do not take into account the EMC issues. The latter will be instead considered in Section 5, which is devoted to the presentation of some results in realistic environments.

### 4.1. Breath Rate Detection

The core of the signal processing algorithm consists of the acquisition of an elaboration matrix **R** that collects a number of received waveforms, comprehensive of the useful signal (*i.e.*, reflected from the thorax) and undesired ones (due to fixed obstacles in the environment, thermal noise, disturbing signals from electronic apparatus—here neglected for the reasons explained in the premise—, *etc.*). More precisely, each row of **R** contains the samples of a received waveform. The time-axis along each received waveform is termed “fast-time” (τ) and has a rather small sampling time *T_f_* (e.g., *T_f_* = 10 ps). The interval between subsequent acquisitions (that is, two adjacent rows of **R**) is indicated by *T_s_* and is in the order of 0.1 s. The corresponding time-axis is termed “slow-time” (*t*). The duration of the measurement interval is denoted by *T_meas_* and the ratio between *T_meas_* and *T_s_* provides the number of rows of **R**.

The difficulty in achieving small values of *T_s_* and, most of all, of *T_f_* is a critical point of the experimental system since, in the absence of an efficient sampling and real time communication of the sampled data, the performance may degrade. However, if the target is to detect only the breath frequency, the theoretical constraints on the sampling interval can be relaxed while continuing to obtain satisfactory results. The impact is more pronounced as concerns the reconstruction of the entire waveform (if desired) where, however, limited sampling can be partly compensated through the adoption of more refined (and therefore complex) detection techniques.

**Figure 12.**
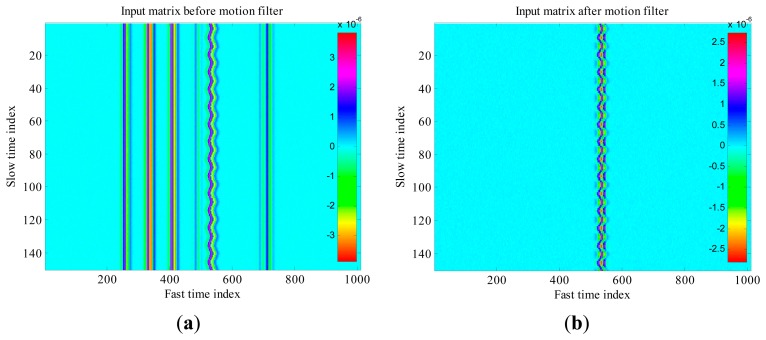
Example of matrix **R**. (**a**) Before motion filtering; (**b**) After motion filtering.

A pictorial example of matrix **R** at the receiver input is shown in Figure 12a: vertical, multi-colored, straight lines denote the presence of static clutter contributions due to some fixed obstacles present in the considered environment; these undesired echoes can be identified and removed through suitable signal processing algorithms.

The static clutter is typically removed by using a motion filter [41] that subtracts from each waveform the average of all the waveforms along the slow-time. Figure 12b represents the same matrix **R** as in Figure 12a, but after application of the motion filter: the static clutter has been completely removed while the oscillating trace, that is due to the thorax reflection and gives the useful signal to detect, is only slightly modified (distorted) by the action of the motion filter.

A spectral analysis can be then realized on the matrix so obtained. An important issue concerns the choice of the fast-time instant at which to perform the spectral analysis. It is possible to show [41] that the best choice for this instant coincides with the propagation time (forward and backward) along the nominal distance *d*_0_ of the subject under test, *i.e.*, τ_0_ = 2*d*_0_/*c*, where *c* is the velocity of light. In some cases the value of τ_0_ is known a priori. This can be considered a realistic assumption for the case of the astronaut during rest.

When this is not, a procedure for estimating the unknown τ_0_ consists of looking at the energy of the motion-filter output and choosing τ = τ̂_0_ such that the energy is maximum. Obviously, in a digital implementation, the accuracy in finding the exact value of τ_0_ is limited by the fast-time sampling interval.

For better evidence, in the following Equations (7) and (8), we represent the received signal as a continuous signal. On the other hand, it should be noted that in presence of a strong oversampling (as required for an efficient estimate) the digital signal can be efficiently described through a continuous waveform. So, noting by *r*(*t*, τ) the signal at the output of the motion filter, supposed to be applied over a 1 Ω resistance (a different value of the resistance would only imply a scale factor, inessential for the analysis), the value of its energy in the interval between *t*_start_ and *t*_start_ + *T*_meas_, is given by:

(7) $$e(\tau )=\int_{t_{\text{start}}}^{t_{\text{start}}+T_{\text{meas}}}{\left|r(t,\tau )\right|}^{2}dt$$

and we must find the value τ = τ̂_0_ such that *e*(τ) is maximum. As an alternative, we can introduce the following definition, that has the meaning of energy of the average value (over the slow-time axis) of the motion-filter output:

(8) $$e'(\tau )=\frac{{\left|\int_{t_{\text{start}}}^{t_{\text{start}}+T_{\text{meas}}}r(t,\tau )dt\right|}^{2}}{T_{\text{meas}}}$$

Potentially, the use of Equation (8) in the place of Equation (7) has the advantage to reduce the effect of the thermal noise, since the latter is averaged through the integral operation. An example in this sense is provided in [42]. An intermediate situation occurs when the value of τ_0_ is known only approximately. Once again, this scenario is significant for the specific application we are considering as, during rest, it is possible that the astronaut makes some limited and slow movements, around the nominal position, that slightly modify the distance between the thorax and the antenna. In this case, a “robust” procedure for counter estimation errors in the local value of τ_0_ consists of computing the Fourier transform not only at the selected τ̂_0_ but also in a range around that value.

#### Example 1

**Figure 13.**
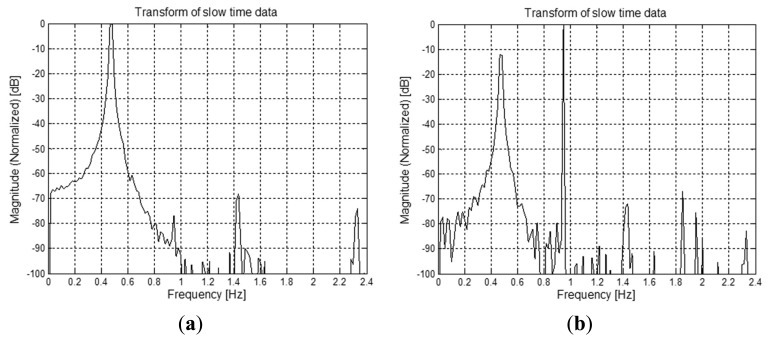
Example of breath rate detection through FFT. (**a**) In the case of (almost) correct τ_0_ estimate; (**b**) In the case of incorrect τ_0_ estimate.

Let us consider a simulated environment, where the thorax movement is modeled through a sinusoidal function (this is a simplifying assumption, that however is acceptable in normal breath conditions. In Section 5, a more general triangle behavior will be also considered) with frequency *f_b_* = 0.475 Hz and amplitude *m_b_* = 12 mm. The nominal distance between the subject and the antenna is *d*_0_ = 0.8 m, that corresponds to τ_0_ = 5.33 ns. Let us consider *T_s_* = 0.2 s and *T_f_* = 10 ps. This setting corresponds to the matrix **R** in Figure 12. Let us suppose that the estimate of τ_0_ provides τ̂_0_= 5.34 ns. The mono-dimensional slow-time FFT computed, at that instant, along the slow-time axis is shown in Figure 13. The Fourier transform shows peaks at the right breath rate (0.475 Hz) and its multiples, so that the unknown quantity *f_b_* can be properly estimated.

**Figure 14.**
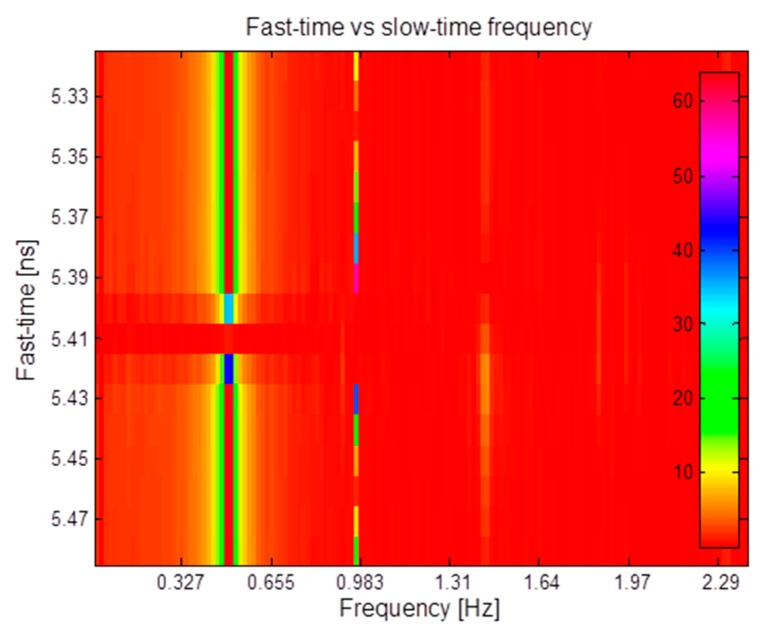
Iterated FFT around the τ_0_ estimate.

**Figure 15.**
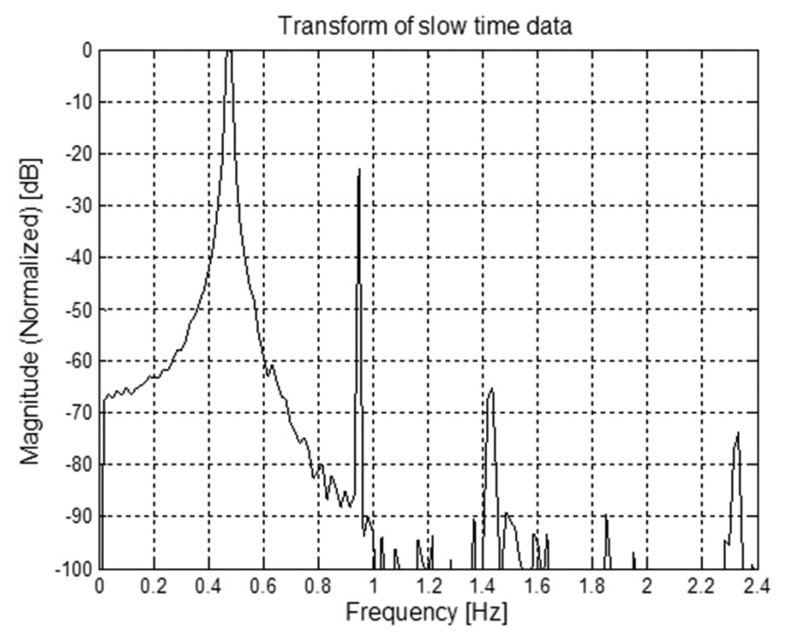
Average FFT for the considered example.

Let us suppose, instead, that the estimate provides a value farther from the exact one, e.g., τ̂_0_= 5.38 ns. In this case, the FFT provides the result shown in Figure 13b. The highest peak now occurs at a frequency that is twice the right value. In conclusion, we can say that the procedure above, based on a single, properly selected, FFT fast-time instant, produces reliable and unambiguous results only on condition that the estimate of τ_0_ is very accurate. As mentioned above, the inaccuracy in the estimate of τ_0_ can be compensated by extending the computation of the Fourier transform to a range centered around τ̂_0_. Figure 14 shows, in a color map, the values of the Fourier transform computed on the set of columns in a range equivalent to ±5 mm around the estimated nominal distance, when τ̂_0_ = 5.38 ns. We see that most of the Fourier transforms, in the considered range of fast-time values, show a peak at *f_b_*. Obviously, coherent with the previous analysis, some transforms have a peak at 2*f_b_*, and this may yield an error if evaluation is done on single vectors. The impact of such wrong estimates is smoothed by averaging the results on the ensemble of transforms. This is shown in Figure 15, that reports the “average” transform so obtained; from the figure, we see that this average spectrum has a peak at *f_b_* that, therefore, can be correctly estimated also in the presence of some error in the estimated value of τ_0_.

### 4.2. Thorax Movement Reconstruction

**Figure 16.**
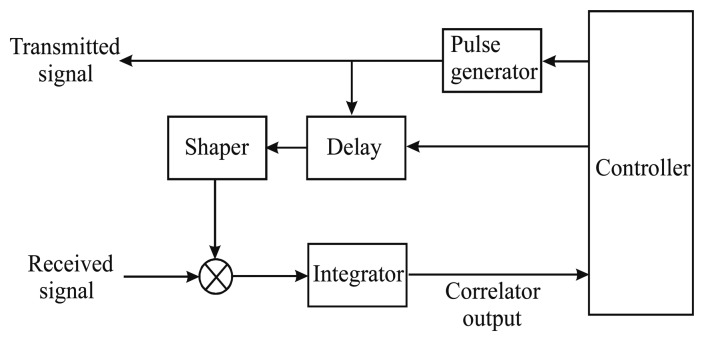
Schematic representation of correlation-based detection.

The accurate reconstruction of the instantaneous thorax position is possible, at least in principle, by using a correlation-based radar. A block scheme of a system of this type is shown in Figure 16. Apart from the possible introduction of suitable scaling factors, the correlation function is defined as the integral (*i.e.*, low-pass filtering) of the product between the received signal *r*(*t*, τ) and a reference signal *s*(*t*, τ). The position of the maximum of the correlation function allows to determine directly the target distance. Computation of the correlation must be repeated at any acquisition, *i.e.*, for any value of the slow-time *t*.

The main problem when using correlation-based methods is the choice of the reference signal. Various options are possible. The simplest choice for the reference signal consists of assuming the same pulse shape as in the transmitter [43]; this, however, makes detection highly vulnerable to possible distortion of the signal. The best correlation, in fact, is obtained when the shape of the reference signal matches well the received pulse (rather than the transmitted one).

For this reason, in [44] a method is proposed that is based on the continuous wavelet transform (CWT). Thanks to the property of wavelets to be localized in time and frequency, they can be adapted, through optimization of the dilatation and translation scale factors, to the received waveform. The main drawback of the method in [44] is the rather long processing time it requires. On the other hand, once having specified the environment, an approximation of the expected received pulse shape can be determined in advance. In the experiment described in [45], for example, a received signal was found, that was very similar to the seventh derivative of the Gaussian function, so that the latter was assumed as the reference.

In [46], we have proposed a different approach, which consists of considering, as the reference signal, the same waveform of the received signal. More precisely, by exploiting the *range-gating* capability that is inherent to the UWB radar, the significant part of the received signal is isolated and then used as the reference.

#### Example 2

**Figure 17.**
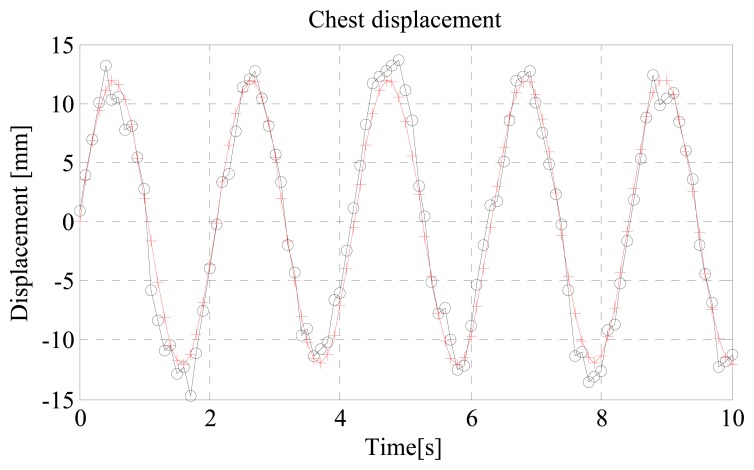
Example of reconstruction of the thorax movement: original waveform (+) against reconstructed waveform (o).

Let us consider the same scenario hinted in Example 1. Following [46], the received signal has been used as the reference signal. The reconstructed thorax movement, originally described through a sinusoidal function with frequency *f_b_* = 0.475 Hz and amplitude *m_b_* = 12 mm, is plotted in Figure 17. Qualitatively, we see that both the values of *f_b_* and *m_b_* are estimated with good accuracy.

The goodness of the thorax movement reconstruction can be measured by computing the correlation coefficient between the original and reconstructed respiration waveforms, defined as:

(9) $$r=\frac{\sum_{i}z_{i}{\hat{z}}_{i}}{\sqrt{\sum_{i}z_{i}^{2}\sum_{i}{\hat{z}}_{i}^{2}}}$$

where *z_i_* is the actual value of the chest displacement at the *i*-th sampling instant and τ̂*_i_* its estimated value. Alternatively, we can compute the normalized-square-error (NSE), defined as:

(10) $$\text{NSE}=\frac{\sum_{i}{\left(z_{i}-{\hat{z}}_{i}\right)}^{2}}{\sum_{i}z_{i}^{2}}$$

#### Example 2 (ctd.)

The normalized-square-error for the waveform shown in Figure 17, where the received signal has been used as the local reference, is NSE = 2.35 × 10^−4^. By using the method in [44] for the same example, the error is smaller, resulting in NSE = 7.18 × 10^−5^. As a drawback, however, the processing time for the method based on CWT is significantly higher (more than 10 times greater). The processing time is also small when using the transmitted waveform as the reference signal but, in this case, the error becomes significantly larger: for the considered example, it is NSE = 1.99 × 10^−2^.

### 4.3. Body Movement Compensation

Obviously, the UWB radar detection techniques is altered by the body movement and, in the absence of proper countermeasures, detecting the respiration waveform and even its rate can become impossible. In spite of its importance, this problem has rarely been faced in previous literature. The solution proposed in [47], for the case of a Doppler radar sensor, adopts a complex signal demodulation technique. The system uses multiple antennas and transceivers to perform detection both from the front and from the back of the human body. Through theory and experiments, the authors demonstrate that a small random body movement (in the order of 5 cm in a time of 5 s) can be completely canceled out. Such a limited and slow movement may be significant when the subject is in rest position (as in the NIMURRA reference scenario), while, in general, wider and faster excursions could be investigated as well. In [13], such an approach is extended by considering a different demodulation method for noncontact vital sign detection, *i.e.*, the arctangent demodulation. In this case, the measurement efficiency is related to the calibration accuracy of the baseband dc offset. A further solution, presented in [48], adopts a differential front-end Doppler radar operating at two different frequencies. All these methods exploit Doppler radar sensors and require at least two antennas.

In [49], we have proposed a method to compensate for the body movement also in monostatic UWB radars, with only one antenna placed in front of the body. The method extends the procedure described in Section 4.1 for the case of still subjects. Similar to the latter, the best fast-time instant, τ_0_, must be identified, at which performing the spectral analysis. As described in Section 4.1 when the subject is still, this can be done searching for the value of τ at which the energy is maximum. In this case the energy can be averaged, for each fast-time instant, over the whole slow-time axis *t*. On the contrary, when the subject is moving, such approach becomes inefficient, and the value of τ_0_ needs to be determined separately for each row (or block of rows, if the subject movement is slow) of matrix **R**. Since τ_0_ measures the “nominal” subject distance *d*_0_, by estimating it we can follow the body movement, compensate it and, finally, recover the unknown parameters.

More precisely, for each row of the matrix, we must estimate the distance between the antenna and the chest, which is influenced by both the respiration activity and the subject movement. In order to assess the respiration parameters, we need to separate these two contributions. For such purpose, we use the estimated distance values as input for a fitting function that interpolates them, as a function of the slow-time, by means of a sum of *m* sinusoidal functions (*m* is the order of the interpolating law). It should be noted that a similar fitting technique is used in [50] to model and cancel the respiration motion when reconstructing the cardiac motion. In that case, however, besides the different target, the authors use a polynomial of order *m* instead of sinusoidal functions. A fitting function suitable for our purposes is available, for example, in Matlab^®^, with 1 ≤ *m* ≤ 8. By adjusting its parameters, the fitting function allows to estimate the subject movement (*i.e.*, the variation of *d*_0_), and to separate it from the breath activity. In principle, separation would be possible by using simple pass-band filters. However, these filters often have overlapped harmonics, which make filtering inefficient. The use of the fitting tool permits us to overcome this drawback. The received waveforms are then realigned by shifting each of them by a quantity that is equal to the estimated subject movement at the corresponding row. This allows to obtain a “compensated” elaboration matrix, which is equivalent to have a still subject placed at the initial distance from the antenna, and to perform a spectral analysis in the same manner as when the subject is still. Moreover, by isolating the chest movement, the value of *m_b_* can be estimated as well. Numerical examples are reported in [49].

**Figure 18.**
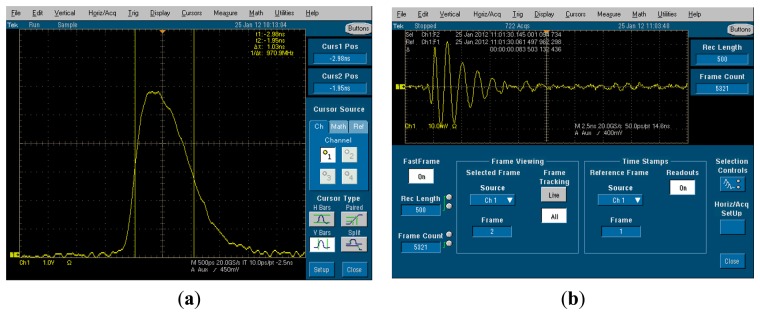
Example of (**a**) transmitted and (**b**) received waveforms in the experimental setup.

In order to assess and validate the considered techniques in a real life environment, an experimental setup has been prepared and a measurement campaign has been carried out. The experimental setup models a bistatic UWB radar exploiting two double-ridge antennas. The equipment involves a Picosecond Pulse Labs 2600C pulse generator (Picosecond Pulse Labs, Boulder, CO, USA) and a Tektronix TDS 7404 oscilloscope (Tektronix, Inc., Beaverton, OR, USA) with digital data acquisition feature, each connected to one antenna. The pulse generator has PRF equal to 1 kHz and pulse length of about 1.5 ns. The oscilloscope has sampling rate equal to 20 GSamples/s and works in “fast frame” acquisition mode. The measurements were performed inside a semi anechoic environment, with anechoic walls placed behind the subject under test, to simulate a low-reflection scenario (like an open environment). Figure 18 reports an example of the transmitted and received waveform in the considered experimental setup.

The experiment was performed by placing a human subject at a distance of about 1 m from the transmitting and receiving antennas and acquiring the backscattered waveforms during an observation time of 60 s. The human subject was breathing normally, with a breathing frequency of about 0.18 Hz.

**Figure 19.**
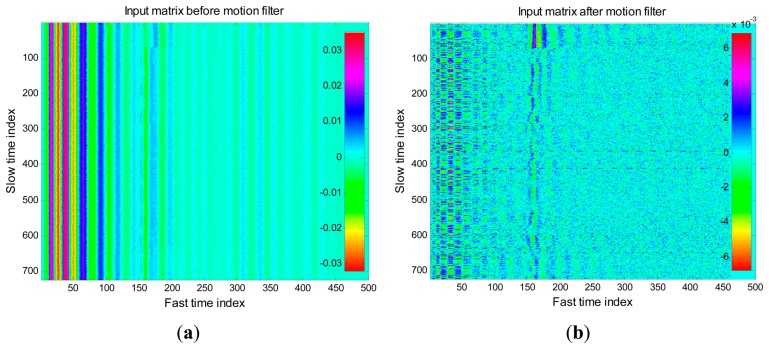
Experimental data. (**a**) Before motion filtering; (**b**) After motion filtering.

The data acquired during this experiment has been processed offline through the algorithms described above. A further correlation-based algorithm has been exploited for the jitter correction, with the aim to compensate for the trigger inaccuracies experienced during acquisitions. Figure 19 reports the matrix **R** obtained from the acquired data, before and after motion filtering.

As we observe from the figure, the sampled data was highly noisy, and affected by high levels of static clutter due to signal reflections (both before and after the transmitting antenna). Despite this, we observe from Figure 19b that the motion filter is able to significantly reduce the static clutter level and thus highlighting a trace corresponding to the breathing signal. The spectral analysis of this trace returned an estimated breathing frequency of about 0.19 Hz, hence the estimation error was less than 6%.

## 5. Application to the Space Environment

The examples presented in Section 4 give an idea of the potentialities of the approach. However, the peculiarity of our analysis is to apply the UWB sensing system to the detection of the breath signal in a complex scenario like that of a space module. For this purpose, it has to be considered that the system works in a highly resonant environment and, moreover, it suffers all the possible EMI signals coming from other sources. The system has to work properly also in this highly noisy environment.

Based on these premises, it is evident that, in order to analyze the performance of the system, a complete characterization of the environment is necessary. The UWB system is intended to be placed inside the racks where the astronauts rest. The choice of locating the system for breathing detection inside the rack where the astronauts sleep was adopted after a long preliminary study concerning the propagation of electromagnetic pulses in the ISS typical environment. The study has permitted us to highlight many critical situations due to multipath [51]. The racks have a metallic structure closed with special fabric for acoustic and solar radiation protection, and a door is placed in the front side of each rack. In order to take into account the presence of the rack, the numerical analysis has been divided in two parts. Firstly, the space module environment and an open rack were analyzed [51]. The results show that, as expected, the reflecting behavior of the module creates a very noisy environment due to the multiple reflections of the structure. The reflected pulses can be considered as a broadband noise superposed to the useful signal coming from the body. It is important to observe that, for the goals of our analysis, it is sufficient to consider, and properly simulate, only the reflections due to the metallic walls of the rack. As mentioned above, the situation would be different if sensing were realized when the astronauts freely move inside the space station; in this case a complete characterization of the multipath profile would be necessary, like that realized in designing a wi-fi network on board of the ISS [52,53]. A sensing of this kind, though certainly interesting, still exhibits several practical problems that make its implementation rather difficult. For this reason, the NIMURRA project has been focused on breath sensing during the rest.

Then, in the second part of the study, the performance of the system was assessed in presence of broadband and narrowband noise sources external to the rack, while considering the human body inside the rack. The effect of the EMI sources could induce errors in the calculation of the breathing frequency, even though their emission levels are sufficiently low to avoid the risk of component damage, as discussed in Section 3.

**Figure 20.**
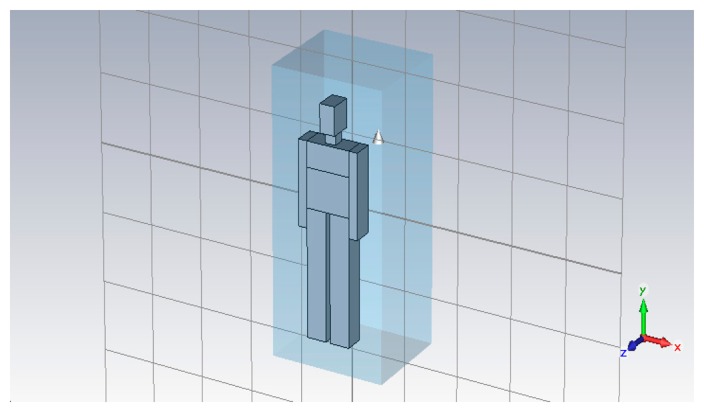
Geometry considered for the calculation of the interfering signal.

The considered geometry is shown in Figure 20: the closed volume has dimensions 104 cm × 85 cm × 201 cm and contains the human body modeled through a phantom. Although highly sophisticated models are available [54,55], the phantom is simulated as if it was formed by a perfectly conductor material, in order to reduce the computational effort. This may seem a strong assumption. Actually, some tests have been carried out to check its reliability: our simulations show that the time domain response does not differ significantly in the two cases of lossy biologic tissues or perfectly conductor material in the first 10 ns [56] which are enough to collect the single measurement, via a proper range gating. Not shown in the figure, the UWB Vivaldi antenna is placed in front of the subject chest.

The respiration waveform is approximated by a triangular behavior (ascending curve for inhalation and descending curve for exhalation). The fundamental frequency is *f_b_* = 0.2 Hz, that therefore represents the unknown to estimate. For processing purposes, the displacement of the thorax during the breathing activity is discretized into different stationary steps. This approximation can be done because the speed of the displacement is very low compared to the pulse speed. The signal to be processed is a combination of all the simulations done for each position of the thorax.

**Figure 21.**
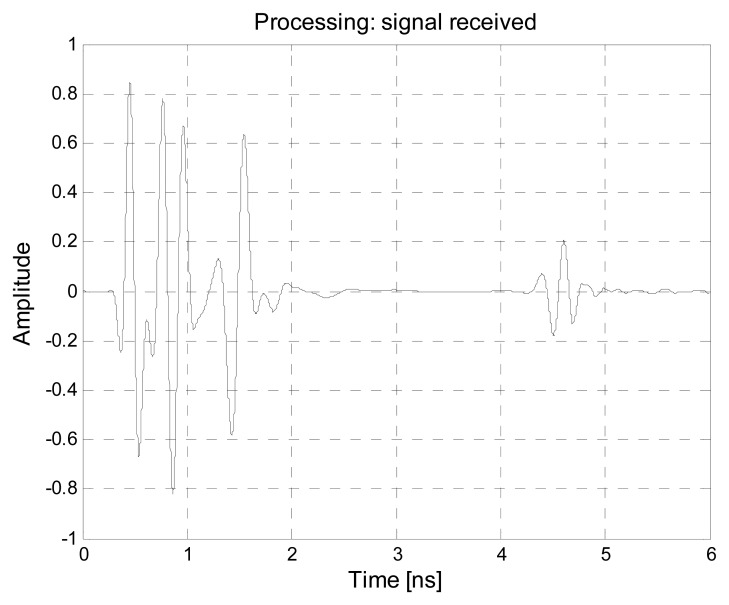
Example of received waveform in the absence of external EMI sources.

**Figure 22.**
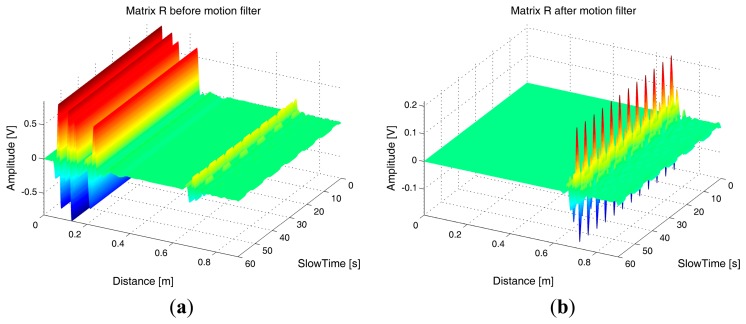
3-D representation of matrix **R** in the absence of external EMI sources. (**a**) Before motion filtering; (**b**) After motion filtering.

Figure 21 reports one of the received waveforms in the considered setting, *i.e.*, by using the subject and rack models, and in the absence of external EMI sources. A set of received waveforms of this kind is collected into the processing matrix **R**, which is reported in Figure 22a. The matrix **R**, so obtained, is used as input for the signal processing algorithms described in Section 4, with the aim to estimate the breathing frequency.

First of all, motion filtering is performed in order to isolate the echoes corresponding to the subject thorax. The result of this processing is reported in Figure 22b, which provides a three-dimensional representation of the matrix **R** after performing motion filtering. We observe that the dominant components are those exhibiting an oscillating behavior during the slow-time, which is due to respiration.

**Figure 23.**
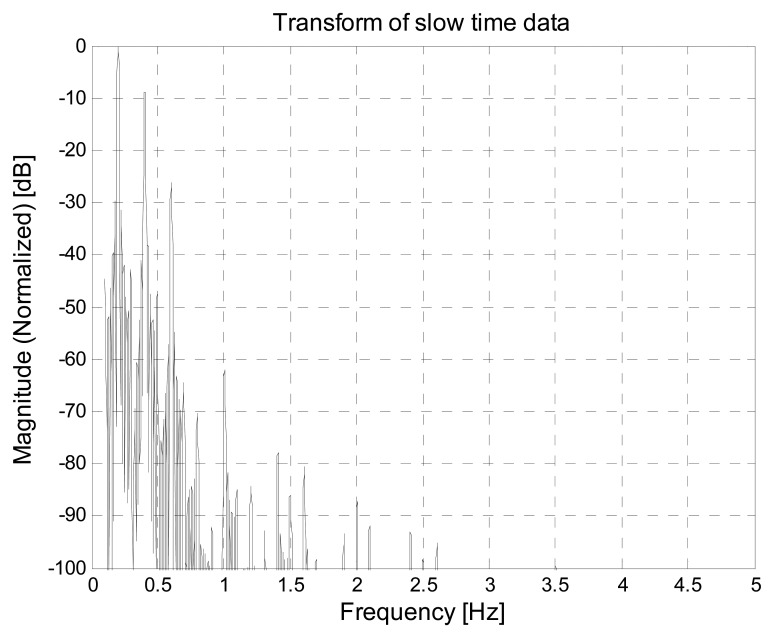
Spectral analysis in the absence of external EMI sources.

This allows to identify the optimal fast-time instant τ_0_, corresponding to the subject distance, and to select the relevant data for the spectral analysis. This way, we obtain the frequency spectrum reported in Figure 23, were we observe that the peaks due to the subject respiration frequency and its multiples are clearly visible. By selecting the highest peak, we obtain and estimated breathing frequency *f̂_b_* = 0.20288 Hz, which is almost coincident with the real respiration frequency in this experiment.

**Figure 24.**
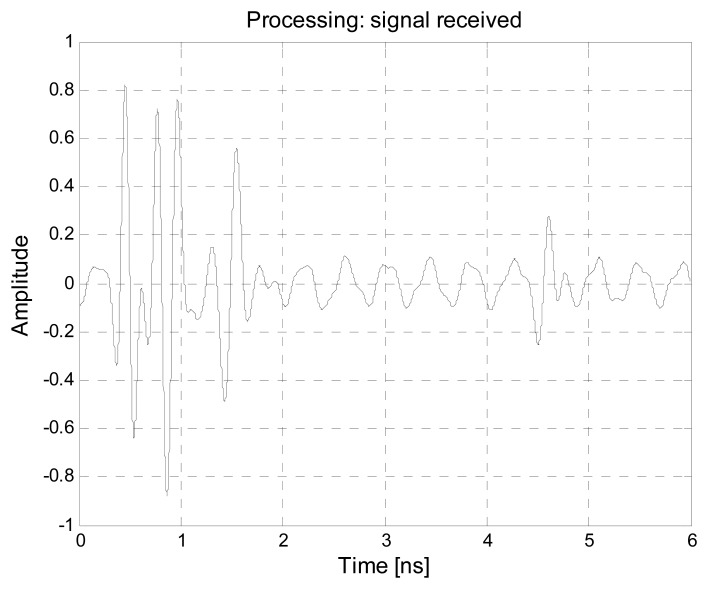
Example of received waveform in the presence of external EMI sources.

Let us now introduce some external sources of EMI signals. For this purpose, an antenna radiating the interfering signals is placed near the human body, simulating a direct illumination between this source and the UWB antenna (worst case). Both a broadband noise signal and a continuous wave signal are considered, radiated by a differential mode source (*i.e.*, a loop) and a common mode source (*i.e.*, a dipole) respectively. In the first case, a loop with a diameter of 1.5 cm is fed with a 4 V trapezoidal clock signal generator having an output impedance of 200 Ω, a frequency of 2 GHz, a rise time of 50 ps and a duty cycle of 50%. This situation could represent the disturbance originated from a small circuit of an electronic device. In the second case, a dipole antenna fed with a continuous wave signal at 2.45 GHz with a voltage generator of 2.4 V is considered. This example can represent the case of a wi-fi system placed near the body of the astronauts. In both cases, the emissions of the disturbing antennas are compliant with the EMC standard inside the module, as specified in Section 3. The thermal noise has been neglected, due to its limited effects compared to the other impairments. The received waveform, which is reported in Figure 24, is obviously more noisy with respect to the previous case, reported in Figure 21.

**Figure 25.**
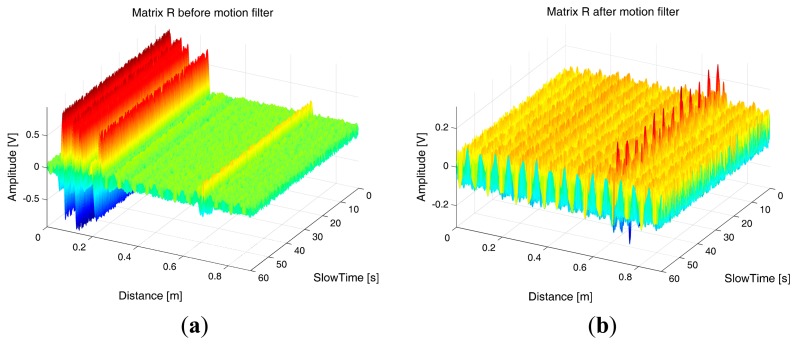
3-D representation of matrix **R** in the presence of external EMI sources. (**a**) Before motion filtering; (**b**) After motion filtering.

Also the matrix **R**, reported in Figure 25a, is now much less regular, and then difficult to analyze. The same matrix cleaned up by the motion filter is shown in Figure 25b.

**Figure 26.**
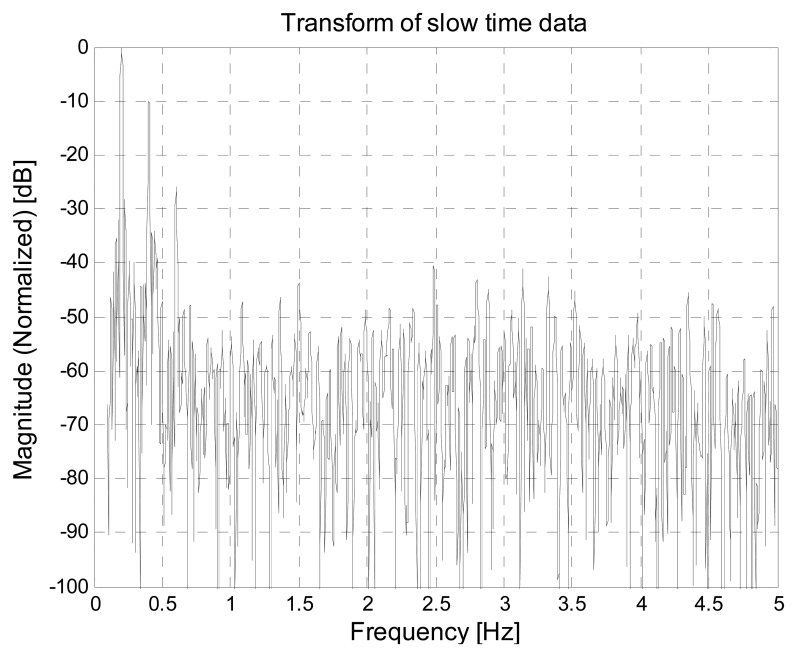
Spectral analysis in the presence of external EMI sources.

Apparently the samples are very noisy; actually, however, the trace of the breathing signal is still clearly visible. Consequently, the spectral analysis provides the curve shown in Figure 26, from which we observe that the peaks corresponding to the breathing frequency and its multiples are still clearly distinguishable. This confirms that, even in the presence of the considered external EMI sources, the breathing frequency can be estimated correctly. In fact, we obtain the same estimated frequency value as before.

## 6. Conclusions

The feasibility study of a system for remote breathing sensing of astronauts was the main goal of the ASI NIMURRA project. One of the main problems faced during the project development was the definition of the system design specifications, because each electronic device on board of the space station has to undergo to regulations much more severe than those addressed to terrestrial environments. In this paper a thorough analysis of the presence of the breath sensor inside the rack where the astronaut rests has been presented. The study was based on simulation of realistic situations as far as the environment characteristics, both in terms of geometry and EMI sources, is concerned. To perform accurate simulations in a critical environment, an experimental validation of the numerical results has been carried out. As the main feature of the system is the contactless sensing through UWB pulses, the radiation of electromagnetic pulses has been analyzed and compared with emission standards in order to assess the most suitable duration, amplitude and waveform of the pulses to be radiated to retrieve respiration frequency. The impact of the electromagnetic environment has been also considered because the highly resonant structure may cause susceptibility problems and malfunctions due to the interference with other electromagnetic sources nearby and with pulse echoes reflected by metallic walls of the rack instead of the thorax movements of the monitored astronauts. The study has also concerned the definition and implementation of suitable signal processing tools able to remove the static clutter from the received waveforms and to isolate the contributions due to the breathing activity. By performing a spectral analysis after an initial filtering, the breathing rate is reliably detected. The whole respiration waveform can also be recovered through the use of a correlation-based approach exploiting a reference signal. The devised signal processing tools have been also validated through an experimental campaign carried out, in the laboratory, on human subjects. Based on our results, we can conclude that the solution based on UWB signals for respiration sensing of astronauts is feasible, robust and it offers sufficient accuracy to highlight conditions of suffering due to lack of oxygen.
